# A Deep-Learning-Based Collaborative Edge–Cloud Telemedicine System for Retinopathy of Prematurity

**DOI:** 10.3390/s23010276

**Published:** 2022-12-27

**Authors:** Zeliang Luo, Xiaoxuan Ding, Ning Hou, Jiafu Wan

**Affiliations:** 1 College of Electro-Mechanical Engineering, Zhuhai City Polytechnic, Zhuhai 519090, China; 2 Guangdong Provincial Key Laboratory of Technique and Equipment for Macromolecular Advanced Manufacturing, School of Mechanical and Automotive Engineering, South China University of Technology, Guangzhou 510641, China

**Keywords:** retinopathy of prematurity (ROP), artificial intelligence, edge–cloud collaboration, deep learning, object detection, telemedicine

## Abstract

Retinopathy of prematurity is an ophthalmic disease with a very high blindness rate. With its increasing incidence year by year, its timely diagnosis and treatment are of great significance. Due to the lack of timely and effective fundus screening for premature infants in remote areas, leading to an aggravation of the disease and even blindness, in this paper, a deep learning-based collaborative edge-cloud telemedicine system is proposed to mitigate this issue. In the proposed system, deep learning algorithms are mainly used for classification of processed images. Our algorithm is based on ResNet101 and uses undersampling and resampling to improve the data imbalance problem in the field of medical image processing. Artificial intelligence algorithms are combined with a collaborative edge–cloud architecture to implement a comprehensive telemedicine system to realize timely screening and diagnosis of retinopathy of prematurity in remote areas with shortages or a complete lack of expert medical staff. Finally, the algorithm is successfully embedded in a mobile terminal device and deployed through the support of a core hospital of Guangdong Province. The results show that we achieved 75% ACC and 60% AUC. This research is of great significance for the development of telemedicine systems and aims to mitigate the lack of medical resources and their uneven distribution in rural areas.

## 1. Introduction

Retinopathy of prematurity (ROP) is a disease of immature retinal blood vessels [1] and is an important cause of vision impairment and even irreversible blindness in premature infants. It mainly occurs in premature infants with low body weight and insufficient gestational age. In a survey of about 15 million premature infants, about 1.2% exhibited ROP, and about 30,000 suffered from permanent visual impairment [2]. The timely screening, identification, intervention and treatment of ROP in premature infants as early as possible are important measures to prevent blindness. Due to their proven efficiency, screening programs for ROP have been increasingly implemented worldwide [3]. However, in China and many other parts of the world, due to the lack of medical resources and medical equipment in some remote areas, this problem still persists, with the main reason being the lack of a sufficient number of ophthalmologists with the necessary professional knowledge and experience [4]. For these reasons, nationwide timely screening and treatment of ROP is not being carried out effectively.

With the development of the fourth industrial revolution, artificial intelligence (AI), communication technologies and Industry 4.0 [5,6] have been developed to a remarkable degree and have seen extensive application. The research on artificial intelligence can be traced back to the pioneering work of Alan Turing, the father of AI, and its aim is to develop systems that can learn and function in a manner similar to humans. Deep learning (DL) models are the most advanced machine learning approaches [7], and their advancement constitutes a substantial global trend. DL models are composed of neural networks that create representations with multiple layers of abstraction to process the input data, and they can perform automatic feature extraction, eliminating the need for manual feature engineering. The process mainly involves the automated projection of low-dimensional data into higher-dimensional spaces. Compared with traditional machine learning technologies, AI and even DL technologies have a large number of real-world applications in many fields, including but not limited to speech recognition, image processing, computer vision, recommendation engines and automatic stock trading.

The most recognized use of AI strategies in retinal disease are the development of spots intricate to disease characteristics on color fundus photos [8]. In the medical field, DL has also led to remarkable results, breaking through the limitations of traditional medical research. For example, it has been successfully applied in the identification of skin cancer, glioma, lymph node metastasis, macular degeneration, diabetic retinopathy, etc. [9]. Before DL, the extraction of the characteristics necessary for the automatic detection of these diseases was a challenging task, and DL technology has provided new ideas and development directions for tackling these challenges in medicine. When it comes to the problem of DL-based ROP detection, Attallah [10] developed an intelligent diagnostic tool based on DL technology using four convolutional neural network (CNN) algorithms to achieve an accuracy rate of up to 93.2%. Wang et al. [11] developed a robotic automatic detection system that is used for the automatic identification and classification of ROP and also designed in parallel two deep neural network models: ID-Net and Gr-Net. The sensitivity of these network models reached 96.62% and 88.46%, respectively, which has important research significance. Peng et al. [12] proposed a novel and effective deep neural network-based five-level ROP staging network, which includes ResNet18, DenseNet121, and EfficientNetB2 as the feature extractors; the results show that this method has good validity and advantages. However, the above algorithm cannot be applied well in practice, so this paper develops a telemedicine architecture based on edge-cloud collaboration, embeds the algorithm into practical engineering applications and achieves good results. The algorithm we propose mainly uses the ResNet101 convolutional neural network and divides the final classification results into four categories. Finally, we integrate the trained model into the Android terminal and use the edge–cloud system architecture to realize the application of the entire telemedicine system.

### 1.1. Contributions

In this paper, a DL automatic identification system based on an edge–cloud collaborative architecture is introduced, and the necessity and significance of the system are discussed in detail. This work’s contributions are multi-faceted:

- First, a detailed overview of the most advanced state-of-the-art DL algorithms is provided, along with their specific applications in the field of ROP detection.
- Second, the necessity of the proposed ROP telemedicine system is discussed in detail, focusing on the edge–cloud collaboration and the DL algorithms discussed in this paper.
- Finally, the relevant medical image datasets used in this study are introduced in detail, along with the specific algorithm development process and the embedding of the algorithm into mobile terminals. Furthermore, future research challenges and development directions in this field are discussed.

### 1.2. Outline

The structure of this paper is as follows. In the second section, the most-mainstream DL algorithms and related applications are introduced; in the third section, applications of our proposed algorithm are presented. In the fourth section, the edge–cloud collaborative architecture system is introduced, and its specific implementation and application are analyzed. The core part of this paper is the fifth section, where the datasets, algorithms, edge–cloud collaborative architecture and the mobile app of the system are introduced. Finally, in the sixth section, a brief overview of the results achieved using the proposed architecture is provided and the potential impact of the ROP telemedicine system studied in this paper is discussed, including its research and social significance as well as future challenges and development directions.

## 2. Deep Learning (DL) Algorithms and Applications in ROP Research

**Figure 1:**
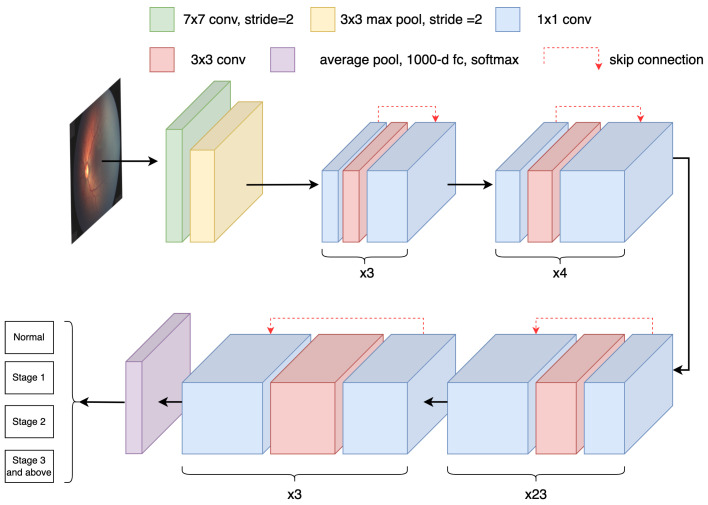
Architecture of a deep CNN.

Deep learning is one of the most important techniques in machine learning [13], with good convergence and generalizability [14], and it mainly involves the use of neural networks to realize low-dimensional mapping of high-dimensional data. The input of the neural network is fed through the input layer and several hidden layers in turn. Each layer learns the intrinsic characteristics of the data through its neurons, and the optimal decision of the multi-layer neuron operation is output from the final layer of the network [15], as shown in Figure 1.

In addition, DL has also achieved excellent research results in the fields of natural language processing, text detection, image processing, speech recognition, remote sensing, medical image recognition, etc. by applying deep learning and processing to information [16,17]. In the following, some outstanding achievements of DL in these fields are presented briefly. Patoary et al. [18] developed a DL model for Bengali language recognition using the Parts-of-Speech (POS) tagging algorithm, and the model achieved a recognition accuracy of 93.90%. Alsukhni [19] constructed a DL model to solve the classification problem of Arabic multi-label text using a multilayer perceptron and a recurrent neural network with long short-term memory. The experimental results showed that the test accuracy in the memory reached 82.03%, while that of the MLP model reached 80.37%. Saba et al. [17] developed a deep learning-based automated system that detects and grades papilledema through U-Net and Dense-Net architectures, which is the first effort in the state-of-the-art for clinical purposes.

In remote imaging research, Li et al. [20] first tried to generate a semantic representation of remote scene categories through a remote sensing knowledge graph representation. A novel deep alignment network with a series of constraints was proposed for cross-modal alignment between visual features and semantic representations, and experimental results showed excellent performance. Zhao et al. [21] adopted a receptive field block net detector, which embedded a receptive field module into a single-shot detector network architecture and obtained higher-level feature representation. The experimental results showed that the algorithm model reached a mean average precision accuracy of 91.56%, which constitutes excellent network performance.

For the analysis of color fundus photos of medical images and coherence tomography angio-graphy (OCTA) [22], the use of artificial intelligence technologies such as DL has developed quite maturely [23]. In medical research, compared with the systematic research of traditional manually defined features, the use of DL neural networks allows effective automatic feature extraction, which not only reduces the complexity of system design but also greatly improves system recognition accuracy, efficiency and precision. To determine possible correlations between different levels of blood pressure (BP) control and retinal microvascular changes in the macula and optic nerve head, Hua et al. [22] used OCTA in hypertensive patients without hypertensive retinopathy. In their research on ROP fundus images, Yildiz et al. [24] developed two datasets with 100 and 5512 posterior retinal fundus images, respectively, and applied them on classifiers such as logistic regression, a support vector machine and a neural network. The extraction and analysis of ROP-related features for the Plus and No Plus categories achieved 99% and 94% Area Under Curve (AUC) accuracy on the two datasets, respectively, showing excellent performance.

Huang et al. [1] trained and applied transfer learning using five neural network models, namely VGG16, VGG19, MobileNet, InceptionV3 and DensetNet. The final experimental results showed that the VGG19 model is superior to other models in the recognition of ROP fundus images, reaching an accuracy of 96%. These studies and findings have promoted the development of DL methods for ROP diagnosis. In addition, Tong et al. [25] trained a 101-layer ResNet CNN and a Faster-RCNN object detection model using 36231 ROP medical images for classification and recognition of fundus images, and ten-fold cross-validation was used for training and optimization. The experimental results showed that the recognition accuracy of the ROP classification reached 90.3%, and the model had extremely high robustness and practicality. Many scholars have made outstanding contributions to the research of DL in the field of ROP identification, some of which are summarized in Table 1.

## 3. Dataset and Proposed ROP Classification Algorithm

### 3.1. Dataset

In this study, 900 color fundus images collected from Guangdong Maternal and Child Health Hospital using a RetCam3 (Natus Medical Incorporated, Pleasanton, CA, USA) in the past five years were used as a dataset; the image resolution was 1200 × 1600 pixels and the dataset included 500 ROP images and 400 normal images. Among them, ROP images were divided into 153 images in the first stage, 239 images in the second stage and 108 images in the third stage and above, and all fundus images were graded by a professional ophthalmologist. In accordance with the provisions and restrictions of the Declaration of Helsinki, the consent of the patients’ guardians were obtained. ROP images have a significant imbalance compared to the 400 normal images. Table 2 describes the division of the training set and the test set.

### 3.2. Proposed ROP Classification Algorithm

**Figure 2:**
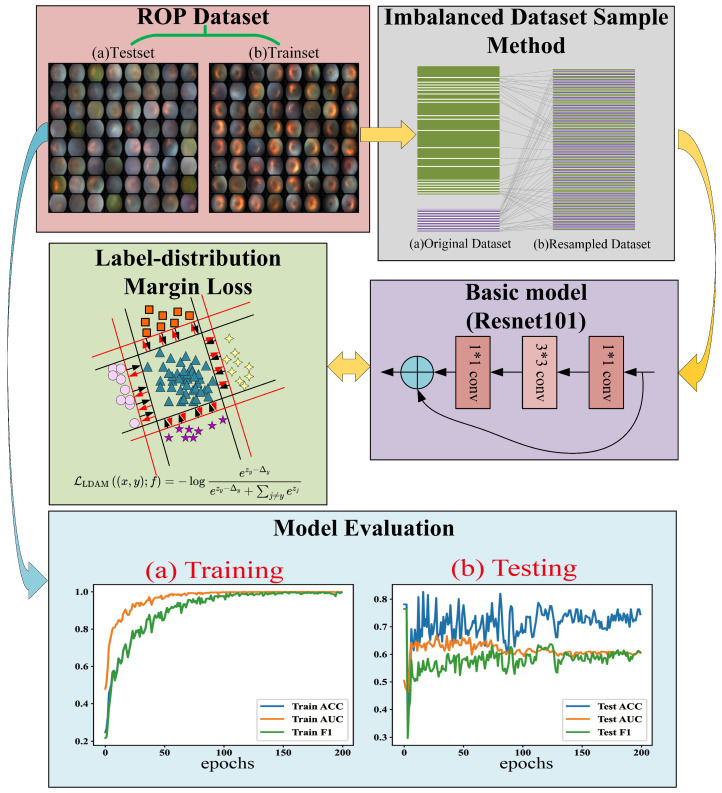
Schematic diagram of our ROP staging networks.

In order to overcome the impact of data imbalance on the diagnosis of neural network models, in the training stage, the data is resampled; that is, the majority classes are undersampled while the minority classes are oversampled. The minority and majority class sampling ratios are the reciprocals of the number of valid samples, which are defined by the following equation:

(1) $$E_{n}=\left(1-\beta^{n}\right)/\left(1-\beta \right)$$

where *n* is the total number of samples for each class and β is a hyperparameter, with 0.999 taken in this paper. Furthermore, Label-distribution Margin Loss (LDML) [29] is introduced to broaden the decision-making space of minority classes and improve the generalization ability of minority classes. The Python OpenCV library is used to preprocess all raw ROP fundus images, including removing the patients’ private information from fundus images. Finally, all images are scaled to a size of 224 × 224 pixels in batches for neural network training based on the Resnet101 model. The process of our ROP staging networks is shown in Figure 2.

**Figure 3:**
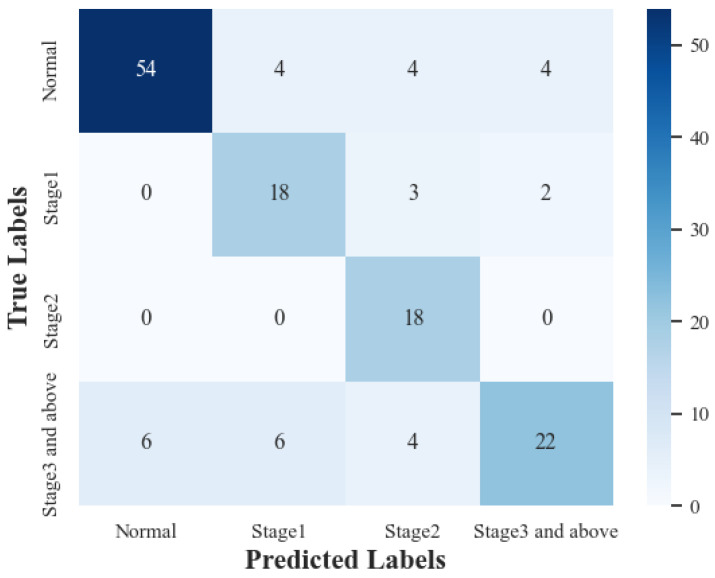
Confusion matrix of our ROP staging networks.

As shown in Figure 2, the accuracy (ACC), area under the ROC curve (AUC) and F1 score (F1) of the training method can gradually converge to 1.0 as the iteration progresses. During the testing phase, ACC gradually converges to 75%, AUC to 60% and F1 to 60%. The confusion matrix is shown in Figure 3. This shows that the proposed training method overcomes the influence of the unbalanced dataset to a certain extent. However, there is still a certain gap in the performance of the current ROC diagnostic model, which is closely related to the characteristics of the small sample and unbalanced dataset used. In the future, the ROP telemedicine diagnosis system based on edge–cloud collaboration architecture proposed in this paper will be further optimized, and the ROP dataset will continue to be amplified to improve the performance of the model.

## 4. Edge–Cloud Collaboration

### 4.1. Introduction

With the substantial increase in the usage of intelligent terminal equipment and the generation of massive heterogeneous data, traditional cloud computing can no longer meet the requirements of some delay-sensitive applications [30]. In the medical and health fields, a series of applications such as computer-aided diagnosis (CAD) and telemedicine have also appeared, placing more stringent requirements on network loads. CAD incorporates multidimensional analysis of medical images, which has great significance in decision making for medical doctors [31]. For telemedicine, it requires patient diagnosis images or allied information to recommend or even perform diagnosis practices while being located remotely [32]. Wan et al. [33] analyzed the development status of biomedical Internet-of-Things-related technologies and pointed out that the demand for lower delays, higher bandwidths, privacy and other aspects have created great challenges to cloud computing. They also proposed that edge computing is one of the pillars of intelligent medical care, which is one of the most feasible methods.

In recent years, more and more studies have proposed edge and cloud computing solutions. Cloud computing along with the Internet of Things (IoT) is proving to be an essential tool for delivering better healthcare services [34]. Edge–cloud collaboration approaches have been fully developed in the medical field and play an important role in the realization of low-latency, low-energy and high-precision computing-intensive tasks [35]. Rahmani et al. [36] defined an intelligent middle layer between sensor nodes and the cloud platform and implemented an IoT-based health analysis system. Aujla et al. [37] proposed a computational offloading scheme for edge–cloud collaboration in order to ensure the quality-of-service requirements of users and verified the superiority of the scheme through calculation of specific performance parameters and a security evaluation of the system. Ding et al. [38] proposed a computer-aided gastroscopic image analysis system based on an collaborative edge–cloud framework that realized real-time lesion localization and fine-grained disease classification in gastroscopic images. Chakraborty et al. [39] designed a framework for integrating body area networks on telemedicine systems based on WBAN, including information gathering, data processing and storing, and monitoring of patients, and the framework provides new ideas for future research in telemedicine.

Within edge–cloud collaboration frameworks, the advantages of the cloud and the edge are complementary and synergistic, and edge devices can utilize the resources of the cloud to supplement their function so as to meet the user’s demand for resources.

### 4.2. Proposed System Architecture

**Figure 4:**
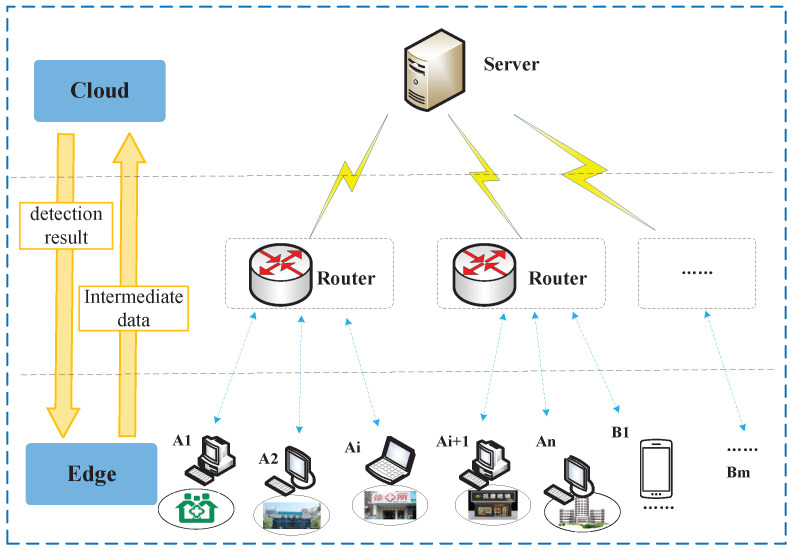
Remote ROP diagnosis system architecture based on cloud–edge collaboration.

In this paper, an ROP telemedicine diagnosis system is proposed based on an edge–cloud collaborative architecture. The system is divided into a three-layer architecture, with a cloud service layer, a network layer and an edge layer, as shown in Figure 4.

The cloud service layer provides data sharing, service sharing, resource sharing and other services for edge nodes. Sample data are collected from each edge service node and are massively processed through a dynamically expandable computational infrastructure using a DL-based ROP intelligent diagnostic model. The network layer guarantees the data transmission for the entire system and mainly relies on various optical fiber, wireless and other communication base stations, while a content distribution network (CDN) arrangement is adopted to improve data transmission speed. The edge layer directly provides ROP screening services to users. A1 in Figure 4 represents edge devices deployed at medical service sites in remote rural areas, while B1 represents edge devices such as mobile and small fundus examination equipment and mobile application clients. The edge layer has data collection capabilities and certain computational capabilities. The network layer connects the cloud server and cooperates with the cloud to form a complete ROP telemedicine system architecture.

During the data collection process, the cloud server can connect multiple edge service nodes. While providing services for multiple places, it can also collect medical record data from various places to enrich its own dataset and thus obtain more comprehensive and accurate lesion characteristics. When the system collects data, the cloud server sends data collection instructions to each edge device, and the edge device returns the response state after receiving the instructions. The cloud sends a random code and stipulates the data transmission protocol with the edge device. Then, the edge device begins to mine the medical record data in the local database, removes the private data and then encodes it, and uploads it to the cloud service according to the agreed protocol.

During the ROP diagnostic process, the edge device collects the original retinal image data of premature infants, performs necessary preprocessing, and transmits the intermediate data obtained to the cloud server through the network layer. The cloud server receives the intermediate data, and the detection results are obtained through the intelligent ROP diagnosis model. Then, the detection results are transmitted to the edge terminal, where users can process them. Compared with traditional cloud-based medical diagnosis systems, the proposed ROP telemedicine system architecture based on edge–cloud collaboration proposed in this paper allocates some of the data processing tasks to edge devices for execution, which effectively reduces the bandwidth pressure of the network and the consumption of cloud computational resources.

## 5. Results and Application

For the design of the ROP telemedicine system, the following modules were implemented: collection of medical image data sets, preprocessing of medical images, ROP diagnosis and analysis, and a mobile application. Using these modules, the complete ROP diagnosis edge–cloud collaborative system was realized, and a telemedicine scheme was successfully implemented. Due to the limitation of the data set itself, the algorithm we adopted cannot get very high precison, but it still has many advantages and availabilities.

First, the medical image datasets were collected from the Guangdong Maternal and Child Health Hospital. Following the regulations and constraints of the Declaration of Helsinki, the consent of the guardians of the patients was obtained. A total of 900 fundus images (including 500 ROP images and 400 normal images) were collected. The Python OpenCV library was used to preprocess all the original ROP fundus images, including removing the patients’ private information from the fundus images and batch-scaling all the images to a size of 224 × 224 pixels for neural network training.

**Figure 5:**
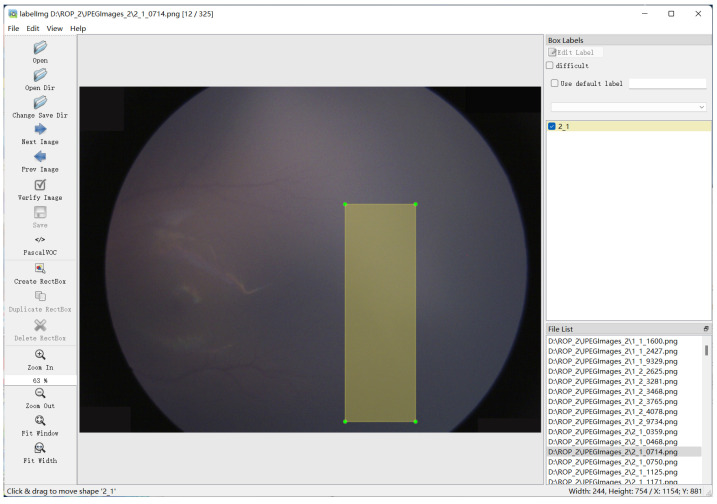
Manual labeling for ROP Images using LabelImg.

Following that, the LabelImg open-source algorithm was used to manually label all fundus images, mark the degree of lesions and whether they were Plus disease, and divide the diseased areas, as shown in Figure 5. Due to the difficulty in obtaining medical images, transformations such as pixel inversion of all pixels, up–down/left–right inversion, Gaussian blur, translation, rotation and contrast enhancement were randomly applied on all images in order to expand the scale of the dataset. Then, the augmented dataset was input into the DL network, and the object detection algorithm was applied to the medical images. This formed the ROP diagnosis and analysis module of the system.

**Figure 6:**
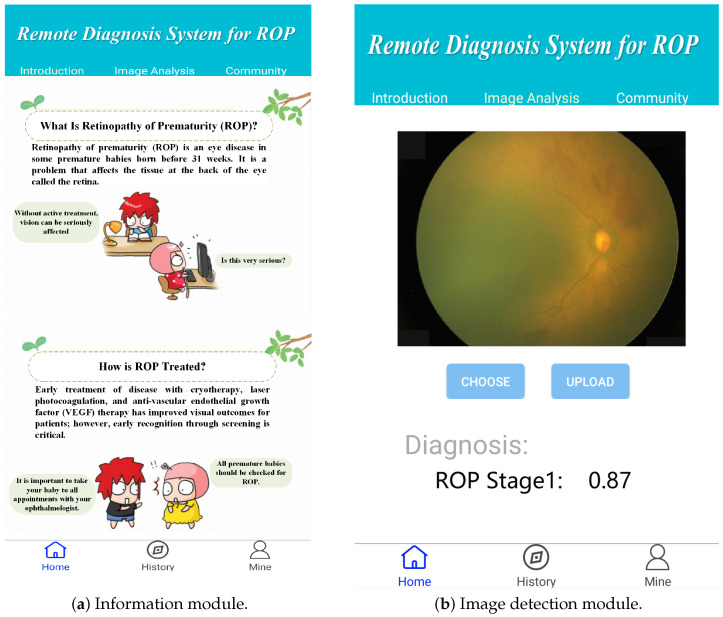
Mobile application interface for the Android system.

**Figure 7:**
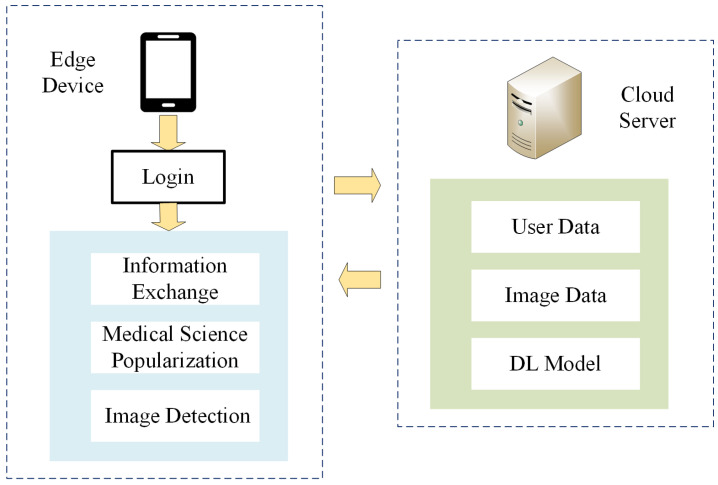
The execution flowchart of mobile application.

In the development process of the edge terminals, we used Google’s Android integrated development tool Android Studio, which has a fast and feature-rich simulator and a large number of testing tools and frameworks to help developers design applications that meet the requirements, to develop a mobile app. We successfully embedded the algorithm mentioned above into the application. The application interface can be seen in Figure 6. The application includes multiple modules, such as an image detection module, a medical science popularization module and an information module, that are installed on the edge devices. The execution flowchart of the mobile application is shown in Figure 7. The diagnostic results of the data are obtained in collaboration with the cloud server and rendered on the detection view. In this manner, a comprehensive edge–cloud collaboration system is formed for remote ROP.

## 6. Conclusions

In this paper, an edge–cloud collaborative architecture system based on DL that integrates a variety of advanced AI technologies for the diagnosis of ROP and the implementation of a telemedicine system is presented. The adopted intelligent ROP diagnosis model based on DL greatly improved the screening and diagnosis ability of ROP. At the same time, the data resampling and undersampling method adopted in this study effectively solved the problem of small ophthalmic medical datasets and led to an improvement of the model’s accuracy and applicability. The results show that we got 75% AUC and 60% ACC. Further, the resource architecture based on edge–cloud collaboration adopted in this study realizes various collaborative methods for resource and data management between the edge terminal and the cloud platform, which ensures that the system can obtain data from multiple sources and improves the robustness, scalability and sensitivity of the system as well as the specificity of the diagnostic models.

The results show that this study can be used to effectively solve the problem of unbalanced medical resources and the lack of professional ophthalmologists in remote areas, and it has high social significance and value. However, the insufficient data leads to low accuracy. For future development, more fundus images will be collected to establish a larger dataset to continuously improve the efficiency and accuracy of algorithm recognition; and we will be gradually improving and developing additional features for the mobile application.

## Figures and Tables

**Table 1:** Several studies on DL techniques in the field of ROP.

Authors	Dataset	Neural Network	Classification	Results
Huang et al. [1]	2452 Images	CNN: VGG16, VGG19, MobileNet, InceptionNet V3, DenseNet	NOROP/ROP, Mild-ROP/Severe-ROP	96% ACC, 98.82% ACC
Wang et al. [2]	52,249 Images	CNN: ResNet18, DenseNet121, and EfficientNetB2	Plus/Normal, Any stage	98.27% AUC, 99.81% AUC
Brown et al. [9]	5511 Images	CNN: U-Net, and Inception V1	Normal/Pre/Plus	91% ACC
Yildiz et al. [24]	5512 Images	CNN: U-Net	Plus/Not-Plus, Pre-Plus/Worse Normal	94% AUC, 88% AUC
Zhang et al. [26]	19,543 Images	DNN: AlexNet, VGG16, and GoogLeNet	ROP/No ROP	99.8% AUC
Hu et al. [27]	2668 Images	CNN: VGG16, inception V2, and ResNet-50	Normal/ROP, Mild/Severe	99.22% AUC, 92.12% AUC
Wang et al. [28]	11,000 Images	CNN: Inception V2, Inception V3, and ResNet-50	Normal/ROP, Mild/Severe	92.7% ACC, 78.5% ACC
Ours	900 Images	CNN: ResNet101	Normal/Stage 1/Stage 2/Stage 3 and above	75% ACC 60% AUC

**Table 2:** Dataset used for training and testing the proposed method in this study.

Class of Stage	Training Set	Test Set	Total
Normal	340	60	400
Stage 1	123	30	153
Stage 2	209	30	239
Stage 3 and above	78	30	108
Total	750	150	900

## Data Availability

Not applicable.
